# Development of Liver Cancers as an Unexpected Consequence of Anabolic Androgenic Steroid Use

**DOI:** 10.7759/cureus.34357

**Published:** 2023-01-29

**Authors:** Sameeha Khalid, Gieric Laput, Kamal Khorfan, Marina Roytman

**Affiliations:** 1 Internal Medicine, University of California San Francisco Fresno, Fresno, USA; 2 Gastroenterology and Hepatology, University of California San Francisco Fresno, Fresno, USA

**Keywords:** hepatotoxicity, cholangiocarcinoma, drug-induced liver injury (dili), hepatocellular carcinima, testosterone hormone

## Abstract

Although the relationship between androgens and hepatocellular tumor development has been noted since 1975, cases involving hepatocellular carcinoma (HCC) or cholangiocarcinoma development in patients on chronic androgen therapy or anabolic androgenic steroid (AAS) use are few, and far between. We present three cases of patients who developed hepatic and bile duct malignancies in the setting of AAS use and testosterone supplementation, arising from a single tertiary referral center. Additionally, we review the literature for the mechanisms behind the possible androgen-mediated malignant transformation of these liver and bile duct tumors.

## Introduction

The relationship between androgens and hepatocellular tumor development was first noted in 1965 in patients with aplastic anemia who were treated with androgen therapy [1]. Since the 1970s the association between oral contraceptives and benign hepatomas, hamartomas, and focal nodular hyperplasia has been widely recognized [2,3]. In the following decades, there have been reports of patients who were treated with androgen therapy for other hematologic, endocrine and rheumatologic conditions who developed hepatocellular carcinoma (HCC) [4-9] and cholangiocarcinoma [10,11]. Most recently a handful of case reports involving young male bodybuilders developing HCC following anabolic steroid use have begun to emerge [12-14]. There have been minimal case reports published regarding the development of cholangiocarcinoma with anabolic steroid use. The development of malignant liver and biliary tract tumors in the absence of chronic liver disease indicates a possible oncogenic disease process mediated by androgens. Albeit the mechanism by which this malignant alteration occurs remains to be elucidated, and the risk remains a serious concern. It is therefore significant to report three cases of men developing hepatic and biliary malignancies in the setting of anabolic androgenic steroid (AAS) use or testosterone supplementation, arising from a single center. We review the literature for the mechanisms behind the possible androgen-mediated malignant transformation of these tumors.

This article was previously presented as a meeting abstract at the 2021 ACG Annual Scientific Meeting on October 22-27, 2021 in Las Vegas, NV.

## Case presentation

Case 1

**Figure 1:**
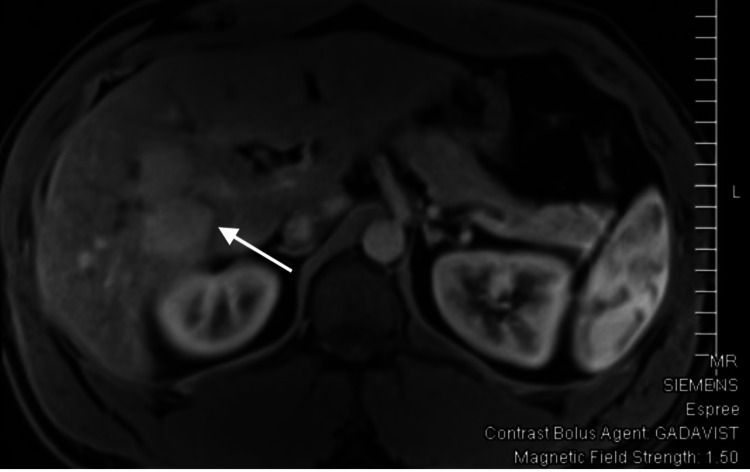
MRI abdomen (Case 1) showing numerous dominant, LI-RADS 5 lesions with arterial enhancement and washout as well as multiple arterially enhancing lesions with variable washout throughout the liver.

A 32-year-old male, a professional bodybuilder, presented to the emergency room with severe right upper quadrant pain. He had been using AAS as well as D-BOL (methadrostenol), a nutritional testosterone supplement marketed toward bodybuilders for four years and had stopped taking both one month prior when routine blood tests revealed elevated liver enzymes. He had no known liver disease or risk factors for it. Physical examination was unrevealing with no notable hepatomegaly or stigmata of chronic liver disease. Laboratory evaluation revealed alanine transaminase (ALT) 46 IU/L, aspartate transaminase (AST) 101 IU/L, alkaline phosphatase 41 IU/L, and total bilirubin 1.2 μmol/L. Tumor markers were within normal limits (alpha-fetoprotein (AFP) level of 1.3 ng/mL and a carcinoembryonic antigen (CEA) level of 0.9 ng/mL). Hepatitis C virus, Hepatitis B virus, hemochromatosis, autoimmune hepatitis, and all other possible causes of liver disease were ruled out. An abdominal sonogram showed no evidence of hepatic steatosis. Computed tomography (CT) scan of the abdomen showed a large, complex, indeterminate mass in the left hepatic lobe and subtle nodular foci in the right hepatic lobe. Magnetic resonance imaging (MRI) of the abdomen revealed 5 dominant, LI-RADS 5 lesions with arterial enhancement and washout as well as numerous arterially enhancing lesions with variable washout throughout the liver (Figure 1). A core needle biopsy of a dominant segment 4 lesion, initially favored hepatic adenoma, showing hepatocellular proliferation with patchy necrosis, with liver plates 1-2 cells in thickness. Additional immunohistochemical stains established the diagnosis of well-differentiated β-catenin, CK-7, and CD-34 positive, HCC. Given the biopsy findings and the extent of multifocal lesions, the patient was evaluated for liver transplantation. Patient underwent transarterial chemoembolization as a bridge to liver transplantation and is currently undergoing phase II liver transplant evaluation. 

Case 2 

**Figure 2:**
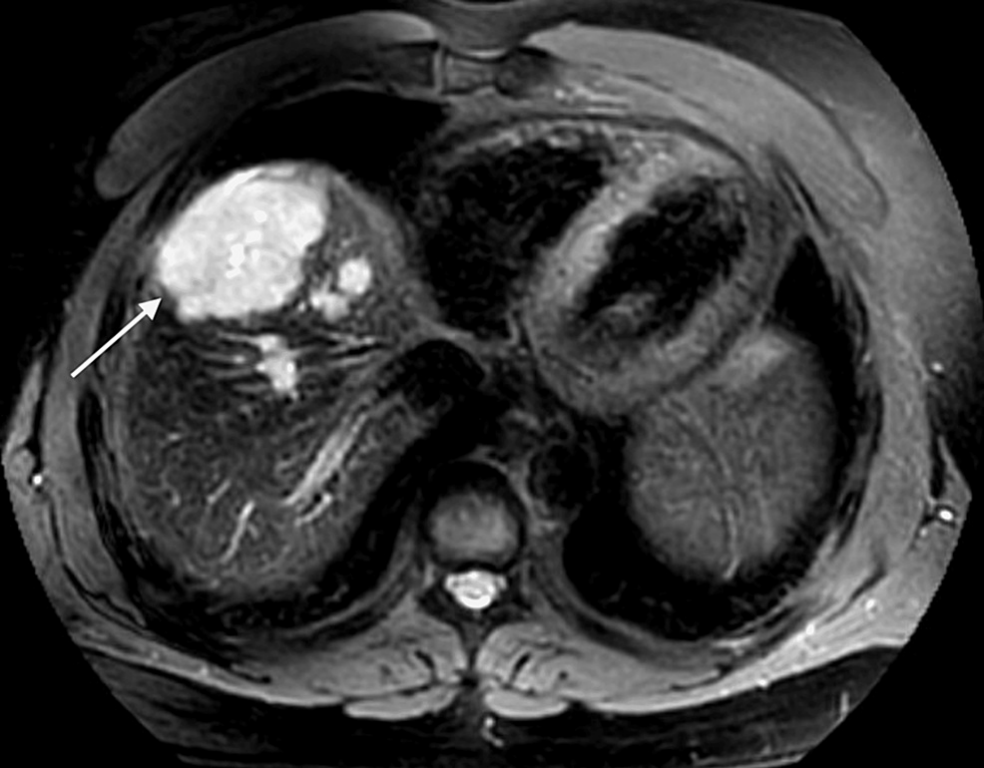
MRI abdomen (Case 2) showing dominant, LIRADS 5 lobulated mass in segment 8 measuring up to 5.6 cm with arterial enhancement with washout and enhancing pseudocapsule with adjacent satellite nodules.

A 55-year-old male with a history of chronically low testosterone levels on testosterone supplementations (via injections previously, most recently via transdermal gel preparation) for over 20 years presented to the emergency room with right upper quadrant pain and found to have a large liver lesion in the right hepatic lobe on CT scan of the abdomen. Laboratory evaluation revealed ALT 42 IU/L, AST 58 IU/L, alkaline phosphatase 69 IU/L, and total bilirubin 1.5 μmol/L. AFP was significantly elevated at 4,326 ng/mL and peaked at >18,000 ng/mL. MRI of the abdomen showed a dominant segment 8 LI-RADS 5 lobulated hepatic mass with arterial enhancement and washout and adjacent satellite nodules as well as additional segment 7 and segment 6 arterially hyperenhancing lesions with variable washout and extensive tumor thrombus in the portal venous system (Figure 2). A liver biopsy was done during initial evaluation at an outside hospital which revealed moderately differentiated HCC. The patient had limited risk factors for liver disease including prior obesity and resolved diabetes mellitus. Laboratory studies ruled out Hepatitis C virus, Hepatitis B virus, hemochromatosis, autoimmune hepatitis and all other possible causes of liver disease. Liver elastography showed no evidence of hepatic steatosis or fibrosis. Testosterone supplementation was discontinued, given the suspicion of linkage to the development of HCC. The patient was started on pembrolizumab; however, he developed hepatic encephalopathy and hyponatremia leading to discontinuation of treatment. He underwent Y-90 radioembolization, with repeat MRI showing favorable response of tumor to Y-90 and alpha-fetoprotein (AFP) decreased to 451 ng/mL from a peak of over 18,000 ng/mL. Two months later a repeat AFP rose to 14,000 ng/mL and CT chest showed new pulmonary nodules concerning for metastatic disease. He was started on atezolizumab and bevacizumab therapy, however continued to have progression of HCC with increasing tumor burden on repeat MRI. He was transitioned to hospice care and died due to disease progression.

Case 3 

**Figure 3:**
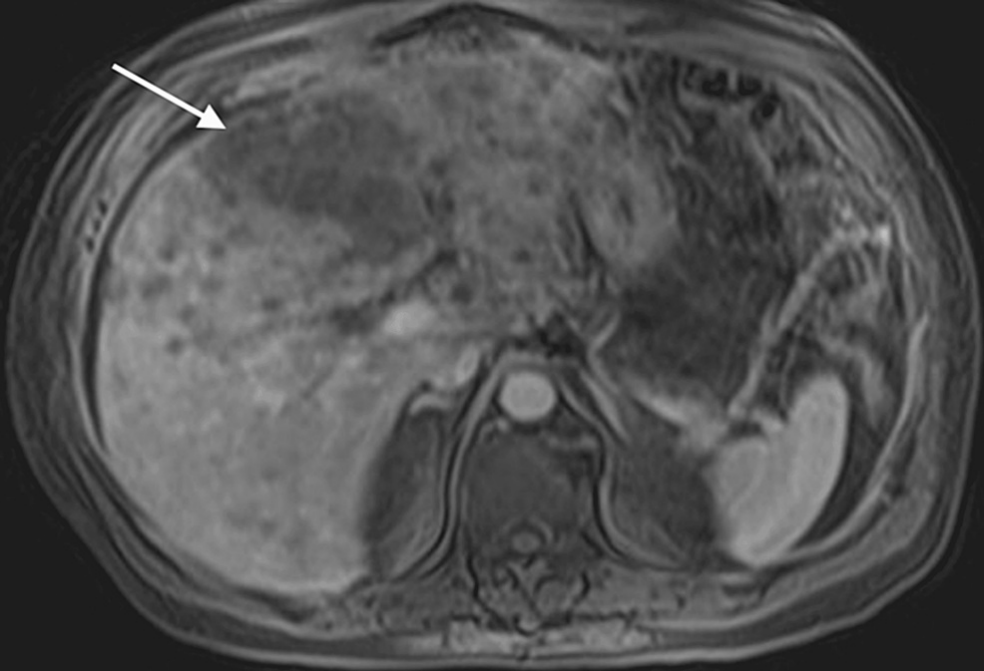
CT abdomen (Case 3) showing large central hepatic mass 8.4x8.4x3.2 cm with numerous hypoenhancing masses throughout liver.

A 56-year old male with a history of hypogonadism on testosterone supplementation for many years presented to the emergency department with one month of worsening abdominal pain. He had recently been evaluated by his primary care provider and had an ultrasound of the abdomen showing an enlarged liver with multiple masses including a 4-cm mass in the right medial lobe. He had no prior history of liver disease and limited risk factors for liver disease, only significant for obesity. Evaluation for Hepatitis C virus, Hepatitis B virus, hemochromatosis, autoimmune hepatitis and other possible causes of liver disease was all negative. Right upper quadrant sonogram showed no evidence of hepatic steatosis. Laboratory evaluation showed ALT 32 IU/L, AST 55 IU/L, alkaline phosphatase 206 IU/L, and total bilirubin 1.1 μmol/L. AFP was within normal limits. CEA was elevated at 126 ng/mL and cancer antigen 19-9 (CA 19-9) was markedly elevated at 13,000 U/mL. A CT of the abdomen demonstrated a large central hepatic mass measuring 8.4 x 8.4 x 3.2 cm with too numerous to count masses scattered throughout the liver (Figure 3). Ascites was present along with peritoneal and omental metastases. A paracentesis was performed with cytology of the fluid positive for malignant cells. CT guided biopsy of an omental lesion was performed with pathology demonstrating a poorly differentiated adenocarcinoma, CK-7 positive, CK-19 positive, consistent with intrahepatic cholangiocarcinoma. The patient continued to deteriorate with the development of sepsis and renal failure with the eventual transition of care to hospice. 

## Discussion

HCC is the most common primary hepatic malignancy and the third leading cause of cancer-related mortality worldwide [13]. The second most common hepatic malignancy is cholangiocarcinoma, known to be an aggressive tumor with a poor prognosis. Our case series of two patients developing HCC and one developing cholangiocarcinoma without any other risk factor besides chronic androgen therapy or AAS is important in bringing higher awareness to these potentially life-threatening complications. 

The hepatotoxic effects of C17-alkylated, 1-methyl and 17-beta-ester testosterone derivatives most commonly used in the United States are well documented [15]. Very few case reports have alluded to a relationship between anabolic steroid therapy and cholangiocarcinoma, mainly citing a temporal but not causal relationship between steroid use and tumor development [11]. Although oral contraceptives have been implicated in the development of hepatic adenomas, only a couple of case reports have linked the use of oral contraceptives with intrahepatic cholangiocarcinoma [16]. 

Cases of malignant transformation of benign hepatic tumors to carcinoma have been reported in the literature [17,18]. A systematic review of case series and reports in 2010 has found that the frequency of malignant transformation to HCC among all adenomas and resected hepatocellular adenomas is between 4.2% and 4.5% [19]. Literature suggests that the type of steroid given affects the benign or malignant nature of the growth [3]. Risk factors of malignant transformation also include a history of androgen or anabolic steroid intake, male gender, history of glycogen storage disease, and mutations of β-catenin. 

Tumors may arise typically after 5 to 15 years of AAS use [12]. Tumor development has occurred more commonly in patients with additional risk factors such as Fanconi’s syndrome, chronic Hepatitis C, or iron overload [12]. These tumors are thought to be well-differentiated and have a better prognosis compared to liver cancers arising in the background of chronic liver disease [14]. 

Malignant transformation

Presence of Dysplasia 

The pathophysiology and genetics involved in the malignant transformation of hepatic adenomas to HCC remain to be elucidated. There is histopathologic evidence of HCC occurring within regions of otherwise typical adenomas, supporting the adenoma-carcinoma sequence hypothesis [10-12]. The classic morphological features of HCC are described as more than two layers of cells in a hepatic plate, pseudo glandular or acinar architecture, and morphologic features including nuclear pleomorphism, and increased mitotic activity [13]. Hepatic adenomas on the other hand have minimal departure from normal liver architecture, consisting of hepatocytes arranged in 1-2 hepatic plates with an intact reticulin framework [14]. However, these distinctions can be challenging especially in certain populations such as those taking oral contraceptives, where hepatic adenoma is more likely to resemble HCC [15]. 

The adenoma-carcinoma sequence hypothesis is reinforced by the idea of “foci of dysplasia” within hepatic adenoma, debated to invariably progress into transformation to HCC [16]. In 1991, Tao first postulated the missing link between oral contraceptive-associated liver cell adenoma and HCC, noting the striking cytologic features of liver cell dysplasia mimicking HCC [20]. Aspirate preparations of liver cell dysplasia within the hepatic adenomas demonstrated a close resemblance to HCC, pointing to a potential mechanism by which malignant transformation may occur [20]. 

Sex Hormone Receptors

With high first-pass metabolism in the liver, AAS has been associated with cases of liver damage and reported to induce significant intrahepatic structural changes. Oxidative stress related to androgen receptor activation has been reported to be the mechanism of AAS-induced hepatotoxicity [21]. Differential expression of wild-type and variant forms of estrogen receptor (ER) and androgen receptor (AR) has been reported in normal liver and HCC, indicating a strong link between sex hormones and the pathogenesis of HCC [22]. HCC is known to be male-dominant cancer and studies have shown that AR rather than androgens play a predominant role in the pathogenesis of HCC [23]. In a study by Zhang et al., it was found that AR is overexpressed in HCC and is associated with disease progression as well as an independent predictor of mortality [23]. In animal models of transgenic zebrafish liver tumors co-induced with oncogenes, sex hormones affected HCC tumor progression and regression with observed sex disparity [24]. Li and colleagues found that androgen promoted HCC progression in females whereas estrogen attenuated HCC progression in males [24]. In a study by Nagasue et al. that assayed HCC tissue and surrounding parenchyma for AR, it found that HCC had a significantly higher concentration of AR than did surrounding liver tissue [25]. Among patients with inoperable HCC, increased variant ER gene expression was previously shown in liver tumors with rapid growth, higher clinical aggressiveness, and lower probability of survival when compared to wild-type ER [26].

Sex hormones have also been linked to the pathogenesis of cholangiocarcinoma. Intrahepatic cholangiocarcinoma cell line HuH-28 expresses ERs [27]. 17β-estradiol enhances protein expression of ERα which modulates neoplastic cell growth and additionally couples with IGF-1R in signaling pathways that promote the growth of neoplastic cells [27]. Androgen-induced hepatic adenomas are relatively rare in comparison to a larger subset of patients having been reported to develop oral contraceptive-induced hepatic adenomas. It is hypothesized that testosterone may play a crucial role in biliary epithelium proliferation, however, this is currently pending further investigation [27]. One study by Yang et al. showed the expression of AR in cholangiocytes and the stimulation of biliary growth by testosterone [28]. Based on this data, patients who develop androgen-induced hepatic or bile duct tumors should discontinue AAS.

Mutations of the β-catenin Gene 

β-catenin and human telomerase reverse transcriptase mutations have been implicated in the transformation of hepatic adenomas to HCC [29]. Similar pathways have been found to play a key role in the induction and progression of cholangiocarcinoma. Steroid use can potentiate malignant transformation of hepatic adenomas via β-catenin mutation occurring as an early event and telomerase reverse transcriptase mutation occurring as the last step. β-catenin (encoded by CTNNB1) is a subunit of the cadherin protein complex that serves as an intracellular signal transducer in the Wnt signaling pathway involved in tumorigenesis [30]. Upregulation in various Wnt ligands along with the redistribution of β-catenin (nuclear translocation) has been seen in the development of malignant biliary tumors [31]. Mutations in CTNNB1 that cause constitutive activation of β-catenin lead to downstream activation of target genes that play a key role in the development of tumors such as hepatocellular adenomas, HCC, cholangiocarcinomas, hepatoblastomas, thus making β-catenin a therapeutic target [30]. The Wnt/β-catenin signaling pathway also plays a role in developing multidrug-resistant cholangiocarcinoma [31]. Surgical resection is indicated in the setting of strong risk factors for malignant transformation including male gender, β-catenin-activated hepatic adenoma, lesion size >5 cm, or any adenomas undergoing 20% or more enlargement or spontaneous hemorrhage [32]. Close surveillance for induction and progression of HCC or cholangiocarcinoma is crucial in patients with a history of congenital or inherited metabolic disorders and the use of estrogens and androgens.

Role of chronic androgen therapy in the development of liver cancers

The data regarding the role of chronic androgen therapy in the development of HCC and cholangiocarcinoma is limited. Yip and colleagues performed a retrospective cohort study to assess the relationship between serum total testosterone and HCC risk in male patients with chronic hepatitis B with diabetes mellitus that found that higher testosterone was associated with an increased risk of HCC (hazard ratio 1.23, 95% confidence interval 1.03-1.46, p=0.024) [33]. Other studies have shown that in patients treated with androgenic steroids, the histological changes found in the liver included bile duct proliferation, peliosis, tumors, and atypical liver cell hyperplasia, supporting the possible causal relationship between androgenic steroids and cholangiocarcinoma [10]. The cases we present here signify that there is a need for additional studies to demonstrate the temporal and causative role of chronic androgen therapy in the pathogenesis of liver cancers.

## Conclusions

Clinicians should have a low threshold to biopsy hepatic adenomas with suspicious imaging findings such as peripheral rim enhancement or contrast washout. Furthermore, on the basis of this strong correlation between AAS therapy and the development of HCC, we propose routine screening for tumor progression in at-risk patients who have been found to have a hepatic adenoma with US imaging and AFP level every six months.

Cases such as these emphasize the need to raise awareness of potentially fatal adverse effects in the medical and bodybuilding communities as well as in patients suffering from hypogonadism, aging men, and patients on long-term estrogen and other steroid therapy. It is critical for clinicians to be vigilant in monitoring patients receiving testosterone supplementation or other androgen and estrogen therapies for potentially life-threatening complications such as HCC and cholangiocarcinoma. Heightened regulation and public health measures are needed in bodybuilding communities to provide education regarding the risk of carcinoma development linked to the use of AAS.

Additional research is needed to better understand the pathogenesis of tumor development in patients on AAS or testosterone-replacement therapy. Future research studies such as prospective cohort studies to observe outcomes of liver and bile duct cancers in subjects taking steroid supplementation may be warranted to better define the relationship between the use of these substances and tumor development. Future directions should focus on the development of universal guidelines to screen for hepatocellular and bile duct cancers in patients on long-term AAS therapy.
